# Parental Home Safety Practices for Domestic Accident Prevention: How Prepared Were Parents for COVID-19 Confinement? A Cross-Sectional Study

**DOI:** 10.3390/clinpract13060129

**Published:** 2023-11-15

**Authors:** Eirini Papachristou, Savas Deftereos, Maria Asimakidou, Konstantina Bekiaridou, Soultana Foutzitzi, Soteria Defteraiou, Panagoula Oikonomou, Ioannis Gogoulis, Christina Nikolaou, Maria Aggelidou, Xenophon Sinopidis, Konstantinos Romanidis, Alexandra Tsaroucha, Katerina Kambouri

**Affiliations:** 1 Department of Pediatric Surgery, Alexandroupolis University Hospital, Democritus University of Thrace, 68100 Alexandroupolis, Greece; epapachri@med.duth.gr (E.P.); asimakidou@yahoo.gr (M.A.); bekiaridounadia@hotmail.com (K.B.); marartem@yahoo.gr (M.A.); 2 Department of Radiology, Alexandroupolis University Hospital, Democritus University of Thrace, 68100 Alexandroupolis, Greece; sdefter@med.duth.gr (S.D.); foutzita@gmail.com (S.F.); 3 Private School “Anodos”, Ainou 23, 68100 Alexandroupolis, Greece; anodosalexpolis@gmail.com; 4 Department of Experimental Surgery, Democritus University of Thrace, 68100 Alexandroupolis, Greece; paoikono@med.duth.gr (P.O.); atsarouc@med.duth.gr (A.T.); 5 Department of General Surgery, Alexandroupolis University Hospital, Democritus University of Thrace, 68100 Alexandroupolis, Greece; ioannis_gogoulis@hotmail.com (I.G.); cnikolaou3@gmail.com (C.N.); kromanid@med.duth.gr (K.R.); 6 Department of Pediatric Surgery, Rio University Hospital, University of Patras, 26504 Rio, Greece; xsinopid@upatras.gr

**Keywords:** COVID-19 pandemic, parents, home accidents, children, injury

## Abstract

(1) Background: Children are susceptible to home injuries. How prepared parents were to protect their children from accidents before and during the COVID-19 quarantine is uncertain. (2) Methods: We conducted a community-based, cross-sectional study in Greece between November and December 2021. We asked parents to complete an anonymous questionnaire voluntarily. Questions focused on accident-preventive measures taken at home during the COVID-19 quarantine. (3) Results: A greater proportion of parents took protective measures for a safer home before the lockdown than during the quarantine, while an interesting percentage of parents never utilized preventive measures for their children. Slightly more than half (58.6%) of parents did not seem to worry about a possible increase in domestic accidents during the quarantine. It was observed that those who had one or two children took more protective measures than those with more than two children. Older parents seemed to explain to their children how to access emergency services more often. Mothers stayed at home with the children more often, and their education affected the presence of some of the assessed measures. In our logistic regression modeling, parental concern about accidents was more related to the incidence of an accident during the pandemic and attendance at seminars. (4) Conclusions: Although the COVID-19 lockdowns disorganized family life, parents were aware of the importance of their parenting role in creating a safe environment for children, but according to the study, there is room for improvement.

## 1. Introduction

Unintentional injuries represent a significant cause of morbidity for children worldwide. Injuries are the leading cause of death in children aged 1–18 years. Many of these accidents occur at home, especially for children under the age of 5 [1]. The COVID-19 pandemic has dramatically changed the lives of people. Government authorities across the world, in order to avoid viral spread and decrease the pressure on healthcare systems, applied uncommon measures, such as social distancing recommendations and school closures. This resulted in more than 2.6 billion people going into a quarantine period [2]. During the pandemic (May 2020), 99% of children around the world lived under some sort of isolation; 60% lived in countries that had a partial or full lockdown, while 1.5 million children did not go to school [3]. This meant that parents and children were forced to spend much more time at home, resulting in a possible increase in domestic injuries.

As the National Action Plan for Child Injury Prevention supports that unintentional injuries are predictable and preventable when precautions are taken, it is evident that many risk situations can only be avoided through parental safety behavior [1]. Parental safety behavior can be subdivided into safety measures taken for a safer environment for the child, education of the child, supervising the child, and giving first aid when an accident has happened [4]. Very little is known about how the quarantine orders affected parental home safety prevention awareness and practices. The only study in the international literature that compares the safety measures parents took before and during quarantine was published in December 2022 by Roberts K et al. [5]. In this study, parents believed that they had made a safe home environment, and there were minimal differences in safety measures or devices used before and during the lockdown period; however, they agreed that during the quarantine, they had the time to identify the areas of their house that needed to be safer, particularly for small children. Since there is not much information about this subject and home safety is a primary reason for reducing domestic accidents, especially in cases of prolonged stay-at-home periods, the present study was designed. This study aimed to examine the preparedness of Greek parents for the prevention of domestic accidents and their behavioral change during the pandemic.

## 2. Materials and Methods

### 2.1. Study Design and Population

We conducted a community-based, cross-sectional, descriptive study via an online questionnaire. The questionnaire was designed by the authors based on safety measures that are described in the international literature; it was extensively tested through a pilot study with 15 volunteers and adjusted accordingly. The study was approved by the Ethics Committee of the Democritus University of Thrace (12216/71-21 October 2021). As this is a cross-sectional study of childhood accidents with an estimated incidence between 16% and 20%, the estimated sample size was 135. The web-based questionnaire was sent to the relevant subjects through widely used parenting websites. We limited the participation criteria to parents of children aged 0–14 who resided permanently in Greece. Moreover, all respondents received the same standardized questions. The purpose of this was to prevent respondents from interpreting the questions differently. Most of the data collected from the questionnaire were operationalized using a different response format (yes/no, Likert-type anchored, and closed-ended questions). This made it possible to process the data quantitatively; therefore, the developed hypotheses could be tested with the use of statistical tools. Between November and December 2021, 140 participants voluntarily completed the questionnaire; subsequently, the study was closed because the strict quarantine had ended in the country during the survey, and while some restrictions still existed, they began to be removed very slowly. We preferred to have opinions that have not been affected by memory loss.

We collected demographic data for both the parents and one of their children (the youngest one), and we asked parents to report the total number of children in their family. The main part of this study was to assess parental preparedness for domestic accidents (10 questions which involved attending first-aid seminars and counseling children as well as suggestions for improving conditions in a future quarantine) and the specific measures taken to prevent accidents at home (28 items assessing the use of different preventive measures for child accidents at home during or before the pandemic—these questions are presented in Table 1). We mandated that the questionnaire be completed by only one of the two parents, and it took about 15 min to complete.

### 2.2. Statistical Analysis

Qualitative variables were expressed as absolute and relative frequencies (N, %). The Chi-squared (χ^2^) test was used to compare percentages among different groups with the use of Fisher’s exact test as appropriate. Continuous variables were expressed as mean and standard deviation. The Mann–Whitney test was used to examine relationships between continuous variables. A binary logistic regression model was used to evaluate the relationship between one dependent variable and a set of independent variables. The individual answers for each prevention measure (Table 2) as dependent/outcome variables were used. These variables were analyzed as either dichotomous (measure before the pandemic/measure during the pandemic) or trichotomous (no measure/measure during the pandemic/measure before the pandemic). Further dependent variables included attendance of an accident prevention education seminar (dichotomous, YES/NO), presence of accident during the pandemic (dichotomous, YES/NO), and frequency that parents talked to their children about accident prevention (scale of 7: never, rarely, sometimes, a few times, often, frequently, always). Parental concern on the increase in accidents during the pandemic (dichotomous, YES/NO) was our main independent variable. Parental and family characteristics were also used as independent variables: number of children in the family (trichotomous, 1/2/>3), paternal and maternal age (either as continuous or dichotomous, >40 years/<40 years), and paternal and maternal education (dichotomous, mandatory/higher). For the logistic regression modeling, parental concern about the increase in accidents as our dependent variable was used. Based on our univariate analysis, the following variables were used as predictors: paternal age (as a continuous variable), maternal education (as a dichotomous variable), presence of accidents during the pandemic (as a dichotomous variable), and seminar attendance (as s dichotomous variable). All statistical analyses were performed using IBM SPSS Statistics version 25.0. Differences were considered significant when *p* < 0.05.

## 3. Results

The basic demographic characteristics of our statistical group are summarized in Table 2.

One father had died, and two others did not want to answer about the age and gender of their child. However, these parents were not excluded from the study because these demographics did not affect their answers about pediatric domestic accidents and safety measures. More than half of all parents 58.6% (N = 82) stated that they were not concerned about the increased risk of domestic accidental injuries during the lockdown. However, many of them advised their children on protective measures either often (N = 49, 35%) or always (N = 42, 30%). In terms of childcare during the lockdown, the majority stated that children stayed at home with either their mother (N = 122, 46.7%) or their father (N = 52, 19.9%). Regarding parental knowledge on the prevention and treatment of children’s accidents, 59.3% (N = 83) claimed to have attended first-aid courses, with 34.9% (N = 29) having a certification in first aid. Only 7.1% of parents attended a seminar or a webinar; this seminar during the above period was organized mainly by either the child’s school (45.5%) or private pediatricians (27.3%). In addition, 82.9% (N = 116) stated that they would like to be trained in first aid. Finally, the majority of parents stated that they have a first-aid cabinet at home to deal with minor accidents (N = 135, 96.4%). We further evaluated the parental motivation to prevent accidents at home by asking parents about the use of specific measures. All questions and parental responses are shown in Table 1.

It is evident that only a few parents took measures specifically during the pandemic. We compared parental concern for accidents during the pandemic between parents that took measures (before or during the pandemic) and those who did not. None of the protective measures assessed provided a difference between the two groups (assessed using the Chi-squared test). When we subsequently compared parents who took measures before and during the pandemic, we found that parental concern for increased accidents during the pandemic only affected Q4 and Q14 (use of non-slip surfaces in the bathroom and use of protective equipment in the terrace). We saw that the parents that were not concerned about accidents took measures in the bathroom (Fisher’s exact test, *p* = 0.053) or used protective equipment in the terrace (Fisher’s exact test, *p* = 0.018) more often during the pandemic than before the pandemic.

The number of children in the family (1, 2, or >3) affected some of the individual measures. In detail, Q2 (use of a child walker), Q10 (presence of poisonous plants), Q25 (availability of fire extinguisher), and Q28 (availability of emergency contact numbers) differed according to the number of children in the family (Fisher’s exact test, *p*1 = 0.05, *p*2 = 0.032, *p*3 = 0.031, and *p*4 = 0.001, respectively) regarding those who took measures and those who did not. We also observed that those who had one or two children took greater protective measures as far as safety covers on sockets (Chi-squared test, *p* = 0.003), checking the water temperature in the bath, and never leaving the child alone (Chi-squared test, *p* = 0.016) compared to parents who had over three children.

Comparing the parental characteristics between the families that took measures before or during the pandemic, we found no relation in either parental age or education level. Maternal education level affected only Q6 (presence of objects that can cause choaking), with mothers of higher education taking measures before the pandemic (Fisher’s exact test, *p* = 0.048). Similarly, comparing the parental characteristics between those who took measures and those who did not, we found sporadic relations. Interestingly, we found that older parents (>40 years) taught their children how to call the emergency services more often (Chi-squared test, for paternal age *p* = 0.035, for maternal age *p* = 0.039). Furthermore, younger fathers were closely related with the non-use of walking aids (Chi-squared test, *p* = 0.048). Younger mothers ensured the use of safety covers for sockets (Chi-squared test, *p* = 0.053) and that rugs were well secured on the floor (Chi-squared test, *p* = 0.043). Overall, paternal education did not affect the existence of protective measures. Interestingly, mothers with higher education (more than the mandatory amount) were the ones that overlooked the use of appropriate safety bars for beds more often (Fisher’s exact test, *p* = 0.018), checking the wear of cables (Fisher’s exact test, *p* = 0.048), and securing heavy furniture and storing heavy objects on lower cabinets (Fisher’s exact test, *p* = 0.048).

We further examined the relationship between the frequency with which parents informed their children and their age and educational level. Only paternal educational level was associated with an increased frequency of advice towards children. Interestingly, it was fathers with only mandatory education that more frequently discussed safety measures with their children (Mann–Whitney test, *p* = 0.007). Similarly, parental perception about increased accident rates during the pandemic and the attendance of safety-related seminars did not affect the frequency with which parents advised their children (Mann–Whitney test, *p* > 0.05 for both). Finally, parents that attended safety-related seminars were the ones that were most concerned about the possibility of increased accidents during the pandemic (Chi-squared test, *p* = 0.014).

As we found that parental concern was related to seminar attendance, we aimed to evaluate if any of the other factors examined would modify this relationship. Using a binary logistic regression model, we assessed other important factors, as shown in Table 3. This model revealed that the occurrence of an accident during the pandemic and the attendance of safety seminars were the only factors associated with increased parental concern.

## 4. Discussion

This study is one of the few studies examining parental safety measures at home before and during the COVID-19 quarantine period [5]. Childhood domestic injuries are perceived as a leading international public health problem since they are one of the preventable causes of pediatric mortality and morbidity [6,7,8]. Many young children are unintentionally injured in their home, a place that everyone believes should be safe [9]. Although child safety is a more complicated situation than we think, a lack of active care, cultural norms, housing conditions, large families, and living in deprived neighborhoods have all been associated with childhood injuries [10,11]. According to the child development literature, a caring, engaged, and constant adult with whom the child can develop a relationship is important [12,13]. In addition, supervision is necessary in a caring relationship, and is beneficial to child safety [9,13,14], and parental adherence is of primary importance for decreasing home accident incidence [15].

The COVID-19 pandemic has compelled families to live in isolation and quarantine, which is likely to affect the well-being of children [16]. During this period with mandatory quarantine in most countries, school and other activity closures, social isolation policies, etc., parents and children spent much more time at home, resulting in an increase in pediatric domestic injuries [17,18,19,20,21]. However, according to the findings of our study, 58.6% of all parents stated that they were not concerned about the possibility of an increased risk of the child having an accident in the house during the quarantine. Quite often (35%), however, parents explained to their children how to recognize the dangers at home and how to protect themselves. This is very encouraging considering that parents’ attitudes and practices are the main components for ensuring a safe home for children’s healthy physical and mental development [15,22]. Previous studies [23,24] showed that 64.8% of parents did not take necessary preventive measures to avoid domestic child injuries, while 53.9% were not informed about the measures required. In our study, only 7.1% of parents had attended a seminar or a web seminar concerning child accidents during the pandemic crisis, which is consistent with the results of a similar survey [15]. It is worth emphasizing that the seminars during the above period were mainly organized by the child’s school (45.5%) or private pediatricians (27.3%). In contrast, in similar surveys, information concerning the prevention of childhood accidents at home came mainly from pediatricians [15,23]. Duan et al.’s study demonstrated that children were more prone to developing smartphone and internet addictions during the pandemic, including through online learning [25]. However, our survey reveals that during this period, some schools and teachers played an important role for the overall promotion of children’s health and well-being by utilizing distanced education and organizing relevant online seminars for parents. Despite this, the percentage of parents who were educated during the pandemic on such a critical issue remains extremely low. It seems that the parents were not properly supported either by the state or private professionals such as pediatricians.

Looking more in depth at the measures utilized before the pandemic, when they were informed about accidents, it is evident from our data that the focus of parental intervention is on the prevention of falls. This could be because it is the most common type of accident in our everyday life [17]. Preventive measures relating to fire or electrocution hazards did not seem to be related to parental intervention as these accidents are less common in everyday life. Interestingly, informed parents that changed their practices during the pandemic focused more on bathroom slips, burn injuries from hot surfaces, and protection from possibly poisonous indoor plants. This probably reflects a shift in parental concerns as our daily lives were disrupted by the pandemic measures.

The majority of all the respondents stated that during the quarantine, their children stayed at home with their mother (46.7%) or with their father (19.9%), which is in contrast with previous search results where grandparents (61.3%) or a babysitter (10.1%) looked after the children at home [15]. This could be explained by the fact that mothers, before the COVID-19 pandemic, due to professional obligations, did not spend much time at home, taking care of their children. Unfortunately, the pandemic disrupted many aspects of daily family life worldwide. Confinement, parental remote working or unemployment, childcare and online education, financial uncertainty, and more increased family stress levels were the hallmarks of this period [26]. In the same study, it was found that the amount of time families spent in front of a screen increased by 74% for mothers, 61% for fathers, and 87% for children [26]. It is evident that parents at home under these circumstances do not seem to protect children from injuries.

Regarding the preventive measures against children’s accidents at home, it was found that only a few parents changed their practice during the pandemic, and the majority of the preventive measures were present before the pandemic. This behavior was related to parental age and education in various ways, depending on the specific measure. We found that age and education (especially maternal) had a role in some of the preventive measures, but not for the majority of them. Doğan and Öztürk also suggested that the risk of accidents may be higher in families with younger parents due to their inexperience and ignorance [27]. Furthermore, Beiki et al. and Ince et al. suggested that injury prevention is less effective among children with low parental education compared with those with higher parental education [6,7]. The finding of our study that maternal education plays more a significant role in accident prevention, although it is not statistically significant in every preventive measure, is consistent with the results of a similar survey, in which a mother with higher education was three times more likely to apply correct practice of injury prevention and first-aid management [6,28].

In the present study, it was also interesting that protective measures were more often taken in families with fewer children (one or two). This could be due to the lack of time in larger households. When parents have to attend to the needs of more children, they subsequently have less time to adhere to all the preventive measures required for their protection. Our statistical group could reflect this situation or possibly highlight a reprioritization in parental adherence to protective measures as these families enlarge.

According to our findings, accident prevention practices by parents, especially during the pandemic, were not particularly satisfactory. Regarding parental knowledge of prevention and management of childhood accidents, it is encouraging that a significant percentage attended relevant events. In addition, 82.9% in our statistical group stated that they would like to be trained in first aid. This fact shows their interest in acquiring knowledge so that they can address possible accidents of their children more effectively. Finally, the majority of parents maintained that they have a first-aid cabinet at home to deal with minor accidents (96.4%). Similar findings were found in a study conducted in Greece [29].

Our summary modeling identified a relationship between parental concern for more injuries during the pandemic, the incidence of accidents during that period, and parental education on the topic. There is arguably a predisposition of parents with children that have more accidents to worry about potential future ones. This could be the reason this relation was evident in our sample. It is somehow a vicious cycle of accidents happening, parents worrying, and eventually seeking advice and training. From this anonymous questionnaire, it is not possible to draw clear conclusions on which of these came first, but further targeted interviews of such families would provide researchers with a better understanding.

Finally, we need to consider the limitations of our survey. Firstly, we should refer to why we used an online questionnaire. It should be noted that since the beginning of the COVID-19 pandemic, researchers have shown an ever-increasing interest in using web-based data collection methods [30] According to Hlatshwako et al., this could be due to the ease of online data collection over traditional face-to-face interviews. Further, it offers a cost-effective and faster way to collect data [31]. This was considered particularly important for our research, as it was our intention to gather our data as quickly as possible so that parents would not forget important details. However, important issues associated with the scientific quality of online survey findings, such as the limited generalizability of online survey samples to the target general population, participant disinterest, or survey fatigue may, have as well influenced our research [32]. In addition, this online questionnaire was written in Greek, with the majority of participants residing in city centers and in urban areas, as filling out an online questionnaire requires a good knowledge of technology and internet access (many villages may not have these). Also, some people might wonder if the time between the end of the lockdown and the completion of these online questionnaires influenced families’ responses due to forgetfulness. However, although the lockdown period in Greece had ended when the study began, many restrictions continued to exist in our country for a long time due to increased cases of coronavirus infections in the community; therefore, the answers were not affected by forgetfulness. Another limitation is that although our sample was adequate to capture the incidence of accidents, it has proved to be too small to capture specific measures and behaviors that changed during the pandemic. Due to the small number of parents in our sample that had taken more preventive measures during the pandemic, it might be that we were unable to capture fine relationships in some preventive measures. Nevertheless, we were able to find some meaningful factors and relationships that explain the situation and are in accordance with the current literature. We also found that the mothers were the predominant people taking care of the children during the pandemic, and their characteristics were related to some preventive measures. This reflects the current situation in our society, in which the mother is the primary carer and the primary person to address household issues. Also, despite the limitations, the major strength of our study should be mentioned. It is one of the two studies that describe the parents’ safety practices during the pandemic, and the first one to do so in Greece. The other one is by Roberts et al. [5]. A multicentric study with different populations should highlight the level of safety at home more in depth, how to avoid child domestic accidents, and how prepared society is, so that in similar periods of confinement, home accidents can be limited.

A very serious issue that was not easy to capture in our study was the association of domestic injuries with possible abusive behavior against children in the domestic environment during the quarantine period. There are conflicting views on whether the incidence of child abuse and neglect increase or decrease during lockdown periods. Some supported that due to the COVID-19 pandemic, anxiety among adults, working from home, fear of possible serious illness, or even the possibility of death are some of the factors that lead carers towards violent behavior against their children [33,34]. However, in a study by Sege R in *JAMA Pediatrics*, 2022, it was reported that the available data showed that there was not a significant change in child abuse and neglect during the lockdown period [35]. In this study, the author expressed his concerns about this finding. Abuse may not have been reported due to closed schools, and admissions to hospitals may not have been made because the injuries were not life-threatening [35]. The same author believed that it was not possible to ask the parents even with an anonymous questionnaire if they abused their children [35]. The same problem was faced in the present study. It was not easy to design questions that could elicit honest responses about whether the injuries involved child abuse. So, we decided to capture parents’ opinions on whether they took or did not take extra safety measures during lockdown to avoid their children’s domestic accidents and to not separate accidents into accidental ones and those due to abuse.

## 5. Conclusions

To our knowledge, this is the first community-based survey of parental knowledge and practices on childhood injuries at home and their prevention during the pandemic in Greece. However, our results indicate that parental efforts to organize a safe environment for their children and protect them from accidents are not enough since children’s accidents at home increased during the quarantine. Based on our results, we would like to provide the following suggestions: As the COVID-19 pandemic begins to subside, parents and the state now have the opportunity to reflect on the lessons learned from the pandemic regarding child accidents, specifically domestic ones. Informing and supporting parents with a view to create a safe home environment for children should be a priority for every society. Governments should invest more resources in injury prevention. Future policies should formulate an action plan to protect children in times of crisis and emergencies. Parents should be able to formulate a similar action plan at home, identifying and improving the safety of their home environment in order to effectively protect their children. It is imperative that both health professionals and all medical structures, public and private, be ready to effectively deal with children’s accidents during times of pandemics and confinement. Pediatricians should be involved more actively in providing injury prevention advice to parents. We should also highlight the importance of information campaigns through the media during such periods, especially for large families, young parents, and parents with a low educational level since, in our study, we observed the most weaknesses in these groups in taking measures to limit home accidents.

## Figures and Tables

**Table 1:** Questions according to specific preventive measures parents took during or before the pandemic.

Measure Taken/Question	N (%)		
	No Action	Measure Taken during the Pandemic	Measure Taken before the Pandemic
Q1. I taught my child how to call the emergency services	93 (66.4%)	3 (2.1%)	44 (31.4%)
Q2. I don’t use a child walker with an unsupervised child	114 (81%)	-	26 (18.6%)
Q3. I have safety gates for the stairs at home	107 (76.4%)	1 (0.7%)	32 (22.9%)
Q4. The bathroom has a non slipping surface or a protective mat	59 (42.1%)	3 (2.1%)	78 (55.7%)
Q5. I never leave the stove or the iron unprotected	37 (26.4%)	5 (3.6%)	98 (70%)
Q6. There are no objects in reach that can cause choaking (i.e., buttons, coins, small toys, nuts)	78 (55.7%)	1 (0.7%)	61 (43.6%)
Q7. I have secured to the wall all heavy furniture. Heavy objects are stored always at the lower shelves	83 (59.3%)	5 (3.6%)	52 (37.1%)
Q8. All sockets have safety covers	46 (32.9%)	5 (3.6%)	89 (63.6%)
Q9. All furniture corners are covered/protected	69 (49.3%)	3 (2.1%)	68 (48.6%)
Q10. There are no poisonous plants at home	66 (47.1%)	3 (2.1%)	71 (50.7%)
Q11. All cables/electricity sources are secured	66 (47.1%)	5 (3.6%)	69 (49.3%)
Q12. I have a CO detector	131 (93.6%)	2 (1.4%)	7 (5%)
Q13. I have no furniture near windows in order to prevent children climbing to the window	89 (63.6%)	6 (4.3%)	45 (32.1%)
Q14. I have protective equipment in the terrace	85 (60.7%)	4 (2.9%)	51 (36.4%)
Q15. I have checked all cables for wearing and there is a safety fusion at home	67 (47.9%)	5 (3.6%)	68 (48.6%)
Q16. I have colourful sticks on the glass windows to make them visible	121 (86.4%)	4 (2.9%)	15 (10.7%)
Q17. I always check the bath temperature and I never leave my child alone in the bath	50 (35.7%)	6 (4.3%)	84 (60%)
Q18. I have removed everything that can cause suffocation from home	86 (61.4%)	3 (2.1%)	51 (36.4%)
Q19. All toys are age appropriate for my children	42 (30%)	6 (4.3%)	92 (65.7%)
Q20. I always lock the entrance door and all windows have safety locks	48 (34.3%)	5 (3.6%)	87 (62.1%)
Q21. I always secure my child to the car seat/stroller	66 (47.1%)	1 (0.7%)	73 (52.1%)
Q22. I have safety bars as appropriate for my children’s beds	73 (52.1%)	2 (1.4%)	65 (46.4%)
Q23. All sharp objects, alcohol, cleaning products and matches/lighters are secured away from the reach of children	72 (51.4%)	4 (2.9%)	64 (45.7%)
Q24. I keep all electric appliances away from my child	65 (46.4%)	5 (3.6%)	70 (50%)
Q25. There is a fire extinguisher in reach at home	97 (69.3%)	5 (3.6%)	38 (27.1%)
Q26. There is a night light in the corridor or the stairs	53 (37.9%)	7 (5%)	80 (57.1%)
Q27. Rugs are well secured to the floor	81 (57.9%)	2 (1.4%)	57 (40.7%)
Q28. Contact numbers for the emergency services are in a visible place	108 (77.1%)	7 (5%)	25 (17.9%)

**Table 2:** Basic demographic data of the cohort.

	N (%)	Mean ± SD
Age of father		43.82 ± 6.12
<40	35 (25%)	
>40	104 (74.3%)	
Age of mother		40.72 ± 5.09
<40	54 (38.6%)	
>40	86 (61.4%)	
Age of child		
0–4 y	44 (31.4%)	
5–9 y	61 (43.6%)	
10–14 y	33 (23.6%)	
Sex		
Male	78 (55.7%)	
Female	60 (42.9%)	
Maternal Education		
Mandatory	10 (7.1%)	
Higher	130 (92.9%)	
Paternal Education		
Mandatory	28 (20%)	
Higher	112 (80%)	
Number of children in the family		
1	56 (40%)	
2	65 (46.4%)	
>3	19 (13.6%)	

**Table 3:** Logistic regression model results.

Variable	Odds Ratio	*p*	95% CI for Odds Ratio
Paternal age (assessed as continuous variable)	0.950	1	0.893–1.010
Maternal educational level (mandatory/higher)	0.887	0.865	0.221–3.561
Accident during the pandemic	2.431	0.021 *	1.145–5.159
Seminar attendance	7.997	0.014 *	1.529–41.623

* indicates statistical significance.

## Data Availability

The datasets generated during and/or analyzed during the current study are available from the corresponding author on reasonable request.
